# Motivational interviewing to promote healthy behaviors for obesity prevention in young adults (MOTIVATE): a pilot randomized controlled trial protocol

**DOI:** 10.1186/s40814-023-01385-0

**Published:** 2023-09-07

**Authors:** Laura N. Anderson, Elizabeth Alvarez, Taylor Incze, Jean-Eric Tarride, Matthew Kwan, Lawrence Mbuagbaw

**Affiliations:** 1 Department of Health Research Methods, Evidence, and Impact, McMaster University, 1280 Main Street W, Hamilton, ON L8S 4L8 Canada; 2 Child Health Evaluative Sciences, Hospital for Sick Children Research Institute, Toronto, ON Canada; 3 Center for Health Economics and Policy Analysis (CHEPA), McMaster University, Hamilton, ON Canada; 4 Programs for Assessment of Technology in Health (PATH), The Research Institute of St. Joe’s Hamilton, St. Joseph’s Healthcare, Hamilton, Canada; 5 Department of Child and Youth Studies, Brock University, St. Catharines, ON Canada; 6 Department of Family Medicine, McMaster University, Hamilton, ON Canada; 7 Department of Pediatrics, McMaster University, Hamilton, ON Canada; 8 Biostatistics Unit, Father Sean O’Sullivan Research Centre, St Joseph’s Healthcare, Hamilton, ON Canada; 9 Centre for Development of Best Practices in Health (CDBPH), Yaoundé Central Hospital, Yaoundé, Cameroon; 10 Division of Epidemiology and Biostatistics, Department of Global Health, Stellenbosch University, Cape Town, South Africa; 11 Department of Anaesthesia, McMaster University, Hamilton, ON Canada

**Keywords:** Obesity, Prevention, Young adults, Health behaviors, Intervention, Motivational interviewing

## Abstract

**Background:**

Obesity is a chronic disease and is an established risk factor for other chronic diseases and mortality. Young adulthood is a period when people may be highly amenable to healthy behavior change, develop lifelong healthy behaviors, and when primary prevention of obesity may be feasible. Interventions in early adulthood have the potential for primary or primordial prevention (i.e., preventing risk factors before disease onset). The primary objective of this study is to determine the feasibility of a 6-month behavioral and educational intervention to promote healthy behaviors for obesity prevention among young adults.

**Methods:**

This is the study protocol for a pilot randomized controlled trial. Young adults (age 18–29) attending McMaster University, Hamilton, Canada, will be recruited and randomized to either the intervention or control. The intervention will include individual motivational interviewing sessions (online or in-person) with a trained interviewer plus educational materials (based on Canada’s food guide and physical activity recommendations). The control group will receive educational materials only. The primary feasibility outcomes that will be evaluated as part of this pilot study include enrollment, retention (≥ 80%), data completion (≥ 80% of weights measured, and surveys completed), and participant satisfaction. Secondary clinical outcomes will include body mass index (BMI) change from baseline to 6 months, physical activity, nutrition risk, health-related quality of life mental health, and economic outcomes. Outcomes will be measured remotely using activity trackers, and online questionnaires at baseline and every 2 months. Risk stratification will be applied at baseline to identify participants at high risk of obesity (e.g., due to family or personal history). Exit questionnaires will collect data on how participants felt about the study and cost analysis will be conducted.

**Discussion:**

Our pilot randomized controlled trial will evaluate the feasibility of an obesity prevention intervention in early adulthood and will inform future larger studies for obesity prevention. The results of this study have the potential to directly contribute to the primary prevention of several types of cancer by testing an intervention that could be scalable to public health, post-secondary education, or primary care settings.

**Trial registration:**

https://clinicaltrials.gov/ct2/show/NCT05264740. Registered on March 3, 2022.

## Background

Obesity is a chronic disease, and it is also a risk factor for other chronic diseases. In Canada, the prevalence of obesity among adults was 28% in 2018 [1, 2]. It has been estimated that the direct costs of overweight and obesity in Canada were $6 billion annually in 2006 [3]. By 2025 approximately 20% of adults worldwide will have obesity.[4] Obesity is defined as a body mass index (BMI) > 30 with classes of obesity severity defined as Class 1 (30–34), Class 2 (35–39), and Class 3 (≥ 40) [5]. Although there are limitations in the use of BMI cut points to define obesity, BMI is strongly correlated with body fat and remains the most practical measure for population-based studies [6, 7]. Obesity severity is important, with more severe obesity conferring a substantially greater risk of cancer, other chronic conditions, and poor mental health [8–10]. Obesity is a complex disease with a broad range of socio-ecological risk factors. Obesity treatment is challenging, and primary and secondary prevention interventions are urgently needed to reduce the incidence of obesity and decrease or maintain obesity severity [11].

The COVID-19 pandemic had a profound impact on activities of daily life, and many causes of weight gain, including chronic stress and physical inactivity, increased during the pandemic. Early indications suggest that 20–48% of people reported weight gain during the pandemic [12–19]. Some reasons include changes in food intake (increased food insecurity and consumption of “comfort” foods), decreased physical activity, increased sedentary time, and increased alcohol. There is some evidence of sex differences (greater weight gain in women) and increased weight gain among people with higher BMI pre-pandemic [12, 13]. One small US study found that pandemic shelter-in-place orders were associated with an average weight gain of 1.5 lbs per month [20]. It is established that obesity can develop slowly over time or as a result of a rapid weight gain [11]. Thus, it is important to investigate interventions that may contribute to modifying health behaviors for obesity prevention.

Young adulthood is a period when people may be highly amenable to healthy behavior change, develop lifelong healthy behaviors, and when the primary or secondary prevention of obesity may be feasible. Interventions in young adults have the potential for primary cancer prevention (i.e., stopping cancer risk factors before they develop). Young adulthood is a time when weight gain may occur rapidly [21] and developing healthy behaviors is critical to maintaining a healthy weight throughout adulthood. On average, Canadian adults gain 0.5 to 1 kg every 2 years and this is greater among young adults and those with higher BMI.[22] Systematic reviews suggest that students attending post-secondary education may experience greater weight gain [23, 24].

Narrowly defined lifestyle interventions for obesity prevention, such as those addressing only physical activity, have had limited success [25]. The 2020 Obesity Clinical Practice Guidelines recommend moving “beyond simplistic approaches of “eat less, move more,” and address the root drivers of obesity” [11]. Yet, there have been relatively few attempts to implement tailored, population-based obesity prevention interventions in young adults. Obesity interventions must be flexible to address the complex causes of obesity, and motivational interviewing may be a successful strategy [25]. Motivational interviewing (MI) is defined as “a collaborative conversation style for strengthening a person’s own motivation and commitment to change” [26]. Health care professionals have successfully used motivational interviewing for patients to set personalized goals for successful healthy life changes [27]. Motivational interviewing has shown some success for weight loss interventions in young adults, [25] but it has not been widely implemented for obesity prevention. It is unknown whether obesity prevention interventions are more successful among people at highest risk. Several obesity risk stratification tools have been developed and validated that identify young adults who are at greatest risk of obesity later in adulthood [28–30]. The use of these risk prediction tools to identify young adults who may benefit the most from an obesity prevention intervention may improve success and support broader population-level, precision public health or clinical interventions.

The primary study objective is:

1. To determine the feasibility (enrollment, retention, data completion, satisfaction) of a 6-month behavioral and educational intervention to promote healthy behaviors for obesity prevention among young adults.

The secondary objectives are:

- 2) To determine the effects of the 6-month behavioral and educational intervention, compared to an educational intervention only, on change in BMI, health behaviors (nutrition, physical activity, and sedentary time), health-related quality of life, and mental health (depression and anxiety).
- 3) To explore whether obesity risk stratification tools identify young adults who may be more successful in an obesity intervention.

## Methods

### Study design and population

We will conduct a pilot randomized controlled trial (RCT). The protocol is reported following the SPIRIT guidelines for RCT pilot studies, [31] the Template for Intervention Description and Replication (TIDieR) checklist and guideline, [32] and has been pre-registered (ClinicalTrials.gov Identifier: NCT05264740). Study participants will be young adults (age 18–29) who are students at McMaster University, Hamilton, Ontario, Canada. Recruitment posters will be posted in common areas on campus, shared on social media, and through email lists. The study website is available at https://motivate.healthsci.mcmaster.ca/.

The study inclusion criteria are:

- English speaking and capable of providing informed consent.
- McMaster University students 18–29 years of age.
- BMI of at least 18.5 (BMI < 18.5 is considered underweight and will be excluded).
- Access to wireless Internet (WiFi) at home.

Exclusion criteria are:

- Physical and mental health conditions that would be contraindications for a weight management intervention, including eating disorders, pregnancy, cancer, or medications that affect body weight.

### Randomization

Participants will be randomized to intervention and control arms in a 1:1 allocation ratio using a computer-generated randomization list that will be generated centrally by the Biostatistics Unit at St Joseph’s Healthcare Hamilton. Randomization will be stratified by sex and BMI (BMI < 25 vs BMI ≥ 25) to achieve some prognostic balance. We will use permuted blocks of random sizes to ensure equal numbers in each group. The block sizes and allocation will be concealed from the participants and trial staff, and the randomization codes will only be revealed after the participants have been recruited and baseline data has been collected. This is an open-label trial. Trial staff and participants will not be blinded to the intervention, but data analysts will be blinded.

### Consent and ethics

Ethics approval was obtained from the Hamilton Integrated Research Ethics Board on July 25, 2022 (HiREB Project Number: 14675). Informed consent will be obtained from all participants at the first in-person visit. Participants may choose to withdraw from the study at any time and when possible, we will capture the reasons for withdrawal. Consideration has been given to issues of equity (advertisement and recruitment materials, research staff and investigators), and among this population of students, we do not anticipate that the English-language requirement will be a barrier.

### Timing of visits, intervention, and control groups

**Fig. 1:**
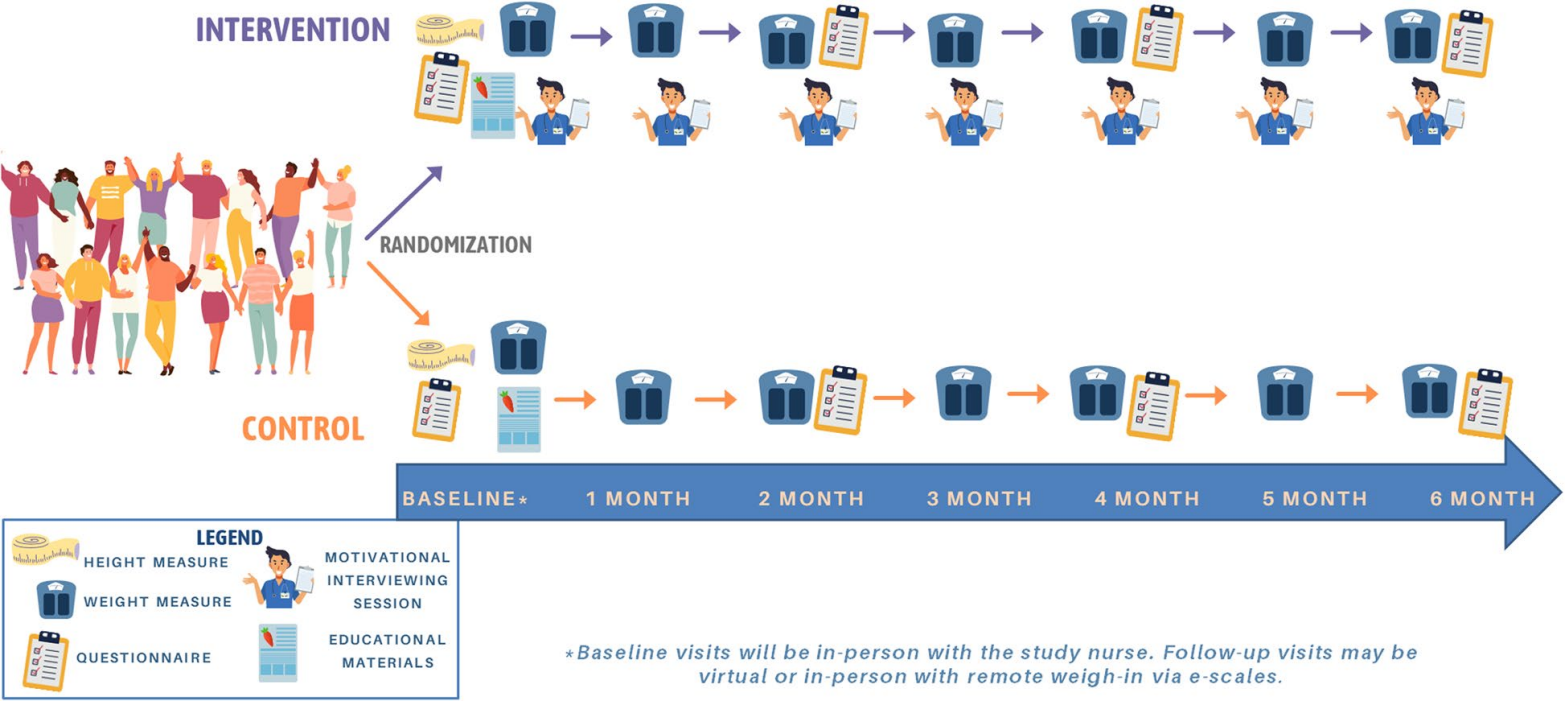
Overview of the study design

An overview of the study design is provided in Fig. 1. The first visit will occur in-person with a research assistant to obtain consent, collect baseline measures, provide participants with the study materials/equipment, and build a relationship with the study participants. For all subsequent visits, participants will have the option of in-person or virtual sessions.

The intervention will include motivational interviewing sessions with a trained research assistant/interviewer plus educational material. The motivational interviewing sessions will occur at baseline and once a month (7 visits in total). For this feasibility study, a priori criteria for the discontinuation or modification of the allocated intervention are not available.

The research assistant will receive training in motivational interviewing. Intervention fidelity (adherence to the principles of MI) will be assessed using recorded training interviews and established rating scales with continuing training as needed [33]. For training purposes, mock interviews conducted between members of the research team will be recorded and evaluated. Interviews with study participants will not be recorded.

**Fig. 2:**
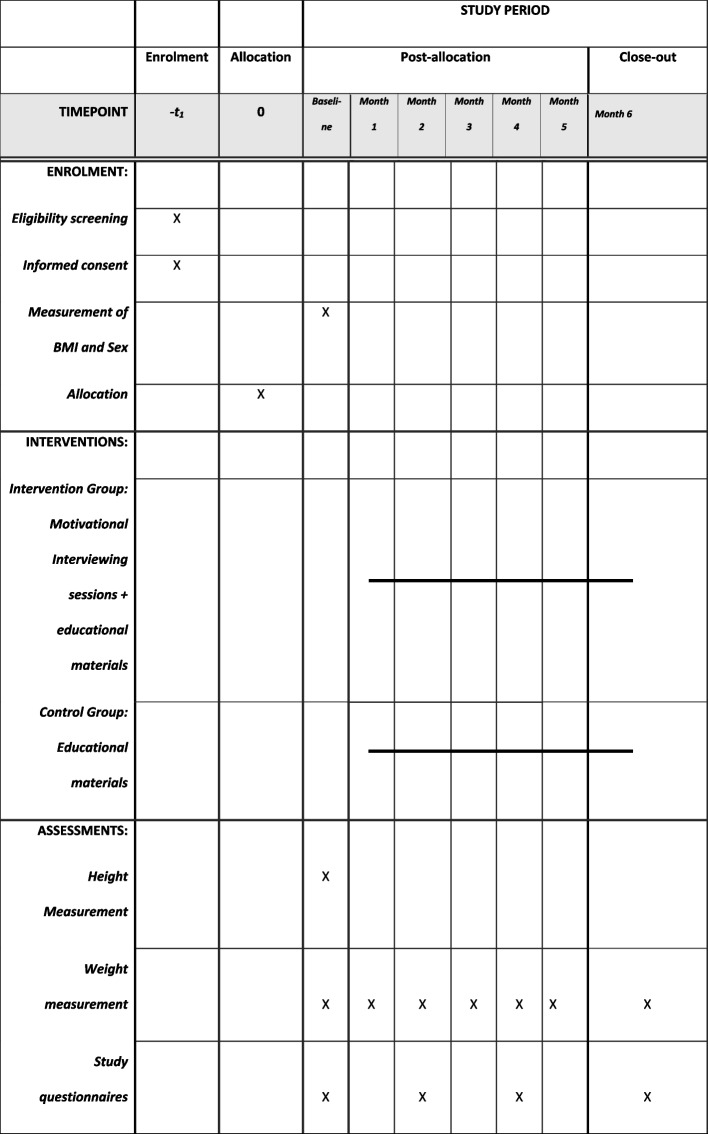
SPIRIT schedule of enrolment, interventions, and assessments

The control group will receive educational material only. The same educational material will be provided to both the intervention and control group and will consist of evidence-based recommendations based on Canada’s food guide [34] and Canadian 24-h movement guidelines [35]. The SPIRIT schedule of enrolment, interventions, and assessments for this study is provided in Fig. 2.

### Measurement of outcomes

Table 1 describes all outcomes. The *primary feasibility outcomes* that will be measured are recruitment, retention, data completion, and participant satisfaction. We will consider a larger trial feasible if recruitment rates are ≥ 50%, retention rates are ≥ 80%, and the data completion rate for all secondary clinical outcomes is ≥ 80%. Participant satisfaction with different aspects of the study will be measured at the exit questionnaire using a Likert scale and semi-structured qualitative free-text responses.

**Table 1:** Summary of all outcome measures and method of analysis

Outcome measure	Variable type	Measurement	Method of analysis
**Primary feasibility outcomes**			
Recruitment rate	Binary	% of eligible participants who are recruited from all those who contact the research team to learn about the study	Feasibility threshold of ≥ 50%
Retention rate	Binary	% of participants who complete 6-month follow-up	Feasibility threshold of ≥ 80%
Data completion	Binary	% of secondary outcomes with no missing data	Feasibility threshold of ≥ 80%
Satisfaction	Continuous	Mean score on a 7-point Likert scale	Mean satisfaction score ≥ 4
**Secondary outcomes**			
BMI change	Continuous	Change in BMI from baseline to 6-month follow-up, weight measures using wifi enabled smart scales	Mean, SD
Physical activity	Binary	International Physical Activity Questionnaire (IPAQ)	Mean, SD
	Continuous	Minutes per day from activity trackers	Mean, SD
Sedentary time	Binary	International Sedentary Assessment Tool (ISAT)	Mean, SD
Nutrition	Continuous	National Cancer Institute’s Dietary Screener Questionnaire (DSQ)	Mean, SD
Mental health	Binary	Depressive symptoms (CESD-10) score > 10, Anxiety (GAD-7) score > 10	N, %
Health-related quality of life	Continuous	Quality of Life (EQ-5D-5L)	Median, IQR

*Secondary outcomes* will include BMI change from baseline to 6-months. Interventions are more successful if longer than 4 months in duration [36]. All study participants will have one in-person visit with the research assistant at the start of the study where baseline weight and height will be measured using standardized methods with calibrated study instruments. Participants (both intervention and control) will then receive a Fitbit activity tracker to wear for the duration of the study. Study participants will be asked to visit the study office once a month to conduct a self weigh-in using a Fitbit electronic “smart” scale (e-scale). They will be asked to weigh themselves monthly, and reminders will be sent by email or text message. E-scales are feasible and valid for research conducted remotely and participant adherence to home weigh-ins is high [37–39], and as part of this feasibility study, we will assess the adherence to self weigh-ins when the scale is located in a private study office. Other secondary outcomes will include nutrition risk, physical activity (measured as 24-h movement, including sedentary time and sleep), and mental health collected using online questionnaires at baseline and every 2 months. Nutrition risk will be measured using the National Cancer Institute’s Dietary Screener Questionnaire (DSQ). The DSQ measures the frequency of intake of selected foods that are known to affect risk of cancer and obesity, including fruits, vegetables, whole grains, and sugary drinks. The DSQ has good validity compared to 24-h recalls [40]. Self-reported physical activity, sedentary time, recreational screen time, and sleep will be measured to describe adherence to recommended 24-h movement guidelines using questionnaires previously used in young adults [41], including the International Physical Activity Questionnaire (IPAQ) [42], and International Sedentary Assessment Tool (ISAT) [43]. The Insomnia Severity Index will also be included as a measure of perceived sleep quality [44]. Mental health is an important upstream determinant of health behaviors and is highly associated with obesity risk. We will measure depressive symptoms, anxiety, and health-related quality of life using the following validated tools: Centre for Epidemiologic Studies Depression Measure-10 (CESD-10) [45], Generalized Anxiety Disorder-7 (GAD-7) [46], and Euroqol-5D-5L (EQ-5D-5L) [47] We anticipate that some youth may also raise issues related to alcohol or substance use in the interviews; thus, we have included questions that ask about alcohol, smoking, cannabis, and medication use in the questionnaires. A single-item measure of body satisfaction is included in the study to evaluate any changes over the course of the study [48]. A validated two-item food insecurity screen is also included [49].

For objective 3, we will evaluate whether feasibility and other secondary outcomes differed for participants who were identified as high versus low risk using existing *obesity risk stratification tools.* Three validated risk stratification tools will be applied to the data to explore these differences [28–30]. The Edmonton Obesity Staging System will also be used [50]. The required variables for each of the risk tools will be collected from the baseline questionnaire (e.g., parent obesity, history of child obesity, alcohol, smoking, medical history). Feasibility data to inform costing and economic analyses for a larger scale trial will be collected. In addition to the EQ-5D-5L, self-reported healthcare use, and time missed from school/work will be collected on the surveys. For this pilot study a descriptive cost analysis will be conducted.

### Data collection and management

All data will be stored in a secure REDCap database. Surveys will be collected online using secure electronic data capture in REDcap. The final trial dataset will only be available to select members of the research team (LA, LM, TI). The final dataset will not be made publicly available to protect the confidentiality of participants, but de-identified data could be made available upon request. Baseline surveys will collect detailed data on socioeconomic variables, including age, sex, gender identity, income, and food security, medical history, and the secondary outcomes described above (nutrition, physical activity, sedentary time, mental health). Follow-up surveys administered every 2 months (at 2, 4, and 6 months) will collect updated data on secondary outcomes. Participants in both the control and intervention groups will receive a $30 gift card at the time of each survey completion. We will continue to collect data from participants if they deviate from protocol unless there is withdrawal from the study. To ensure confidentiality, a unique study id number will be used for the weight and activity data and linked at McMaster by the research team to the main study database.

### Safety protocols and unanticipated outcomes

Possible adverse outcomes may include concerns related to mental health, including eating disorders or frustration due to weight gain/no change. Expectations of the research will be laid out before enrollment and feelings such as these can be addressed as part of the motivational interviewing process. Our research team including the research assistants/interviewers and physician co-investigator (EA) are equipped to assess the situation and provide necessary referrals. Medical and counseling services are available on campus for all students. If participants raise any concerns of self-harm or harm to others, then safety protocols will be followed.

### Data analysis

The analysis and reporting of our results will be done according to the CONSORT guidelines [51]. The data analyst will be blinded. Patient screening, randomization, allocation, and follow-up numbers will be illustrated in a flow diagram. Baseline data will be reported in a table for both groups (intervention and control) and summarized as means (standard deviation) or median (first quartile, third quartile) for continuous variables and counts (percent) for categorical variables. Data on feasibility outcomes will be measured and compared to pre-determined thresholds for interpretation. The primary analysis will be by intention-to-treat (data from participants will be analyzed according to their allocation irrespective of whether they received that intervention). Comparison of groups for secondary outcomes will be reported descriptively for this pilot study. No formal significance testing for comparisons of the secondary clinical outcomes will be made because this is a pilot study with a priori sample size determined based on feasibility outcomes only. It is well established that there are sex and gender differences in obesity [52]. Disaggregated data on sex and gender will be reported.

### Sample size

Our sample size estimation was based on feasibility considerations and calculated using WINPEPI [53]. Using a 95% confidence level, assuming that 80% of the participants will complete the study with a 7.5% margin of error, 110 participants are needed (55 intervention and 55 control). Since the randomization will be stratified by sex and BMI (BMI < 25 vs BMI ≥ 25), we will have approximately equal numbers in each group. This sample size is not sufficiently powered to make between-group comparisons but will provide accurate data on feasibility and estimates of effect size variability that will inform the sample size calculation of a larger definitive trial.

### Reporting and interpretation

Study results will be reported following the CONSORT extension for randomized pilot and feasibility trials guideline [54]. For each feasibility outcome, we will report whether the feasibility threshold was met and use a traffic light system to inform progression to a larger trial. Green, proceed to larger trial; yellow, proceed with some modifications; and red, larger trial not feasible. At least one feasibility outcome must be green. Any deviations to the protocol will be recorded and reported in the final manuscript. Study results will be shared with participants through a final report and published in a peer-reviewed manuscript.

## Conclusions

Although it is well recognized that the causes of obesity are complex, many obesity trials have evaluated a narrowly defined “one-size-fits-all” intervention. We will evaluate the feasibility of motivational interviewing sessions that will be flexible and allow participants to discuss the health behaviors that are most important to them, thus tailoring the intervention to their needs. This approach will allow participants to set their own behavioral change goals. Further, we will explore the feasibility of a risk stratification approach to an obesity intervention to determine if higher-risk young adults benefit more from the intervention.

This intervention is designed to be flexible to focus on a broad range of health behaviors so study participants can set behavioral change goals related to the behaviors that they think are most important to them. While for some participants, this may focus on traditional, obesity-related risk factors such as diet or physical activity, for other participants this may include behaviors related to sleep, mental health, substance use, or time management. All these behaviors have the potential to either directly or indirectly impact obesity prevention.

Our feasibility trial will inform future larger RCTs for obesity prevention. The results of this study have the potential to directly contribute to the primary prevention of several types of cancer by testing an intervention that could be scalable to public health, post-secondary education, or primary care settings. This study is pragmatic with the option of remote visits for MI. Unlike traditional MI, this format with a trained interviewer is feasible and the results of the risk stratification objective may inform who would benefit the most from a larger-scale program. This pilot study will provide proof of concept for an intervention that could be expanded to other settings such as public health, post-secondary education, or primary care. If successful, this program could be implemented in university wellness services and may have a profound impact on the primary prevention of an established and highly prevalent chronic disease risk factor.

## Data Availability

Not applicable.

## Abbreviations

BMI: Body mass index
CESD-10: Centre for Epidemiologic Studies Depression Measure-10
DSQ: Dietary Screener Questionnaire
EQ-5D-5L: Euroqol-5D
GAD-7: Generalized Anxiety Disorder -7
HiREB: Hamilton Integrated Research Ethics Board
IPAQ: International Physical Activity Questionnaire
ISAT: International Sedentary Assessment Tool
MI: Motivational Interviewing
RCT: Randomized controlled trial
